# Supplementary material for: Prenatal methadone exposure disrupts behavioral development and alters motor neuron intrinsic properties and local circuitry
Source: eLife. 2021 Mar 16;10:e66230. doi: 10.7554/eLife.66230 (PMC7993998; doi:10.7554/eLife.66230)
Supplement: Supplementary file 1. — n = 3 dams + their respective litters per timepoint; n = 17–20 offspring samples at G18, n = 15 offspring at P1, n = 17–18 offspring at P7. All tissue and blood samples were collected 2.5 hr following the morning administration of methadone. Data are collapsed across offspring sex. All data are mean ± SEM. EDDP: 2-ethylidene-1,5-dimethyl-3,3-diphenylpyrrolidine. The limit of quantification for methadone and EDDP detection was 0.1 ng/mL and 0.05 ng/mL in the plasma, respectively, and 0.08 ng/sample and 0.04 ng/sample of placenta and brain for both methadone and EDDP. [file elife-66230-supp1.docx]

**Supplementary File 1 Dam and Offspring Methadone and Metabolite Concentrations.**

| **Plasma (ng/mL)** | | | | | | | | |
| --- | --- | --- | --- | --- | --- | --- | --- | --- |
|  | **Gestational Day 18** | | **Postnatal Day 1** | | | **Postnatal Day 7** | | |
|  | **Methadone** | **EDDP** | **Methadone** | | **EDDP** | **Methadone** | **EDDP** | |
| **Dam** | 63.5 ± 11.3 | 74.6 ± 22.0 | 21.4 ± 6.3 | | 38.8 ± 5.3 | 16.0 ± 2.2 | 40.4 ± 3.9 | |
| **Offspring** |  | | 0.5 ± 0.1 | | 3.0 ± 0.5 | 0.5 ± 0.1 | 1.4 ± 0.4 | |
| **Brain (ng/g)** | | | | | | | | |
|  | **Methadone** | **EDDP** | **Methadone** | **EDDP** | | **Methadone** | | **EDDP** |
| **Dam** | 248.9 ± 54.1 | 16.8 ± 2.0 | 92.3 ± 17.0 | 16.9 ± 3.0 | | 73.0 ± 10.7 | | 15.9 ± 1.3 |
| **Offspring** | 2100.8 ± 237.6 | 27.6 ± 8.4 | 7.9 ± 0.6 | 1.8 ± 0.2 | | 3.1 ± 0.3 | | 0.01 ± 0.01 |
|  |  |  |  |  | |  | |  |
| **Placental (ng/g)** | | | | | | | | |
| **Placental** | 3862.1 ± 258.4 | 1124.0 ± 84.4 |  | | | | | |

n=3 dams + their respective litters per timepoint; n=17-20 offspring samples at G18, n=15 offspring at P1, n=17-18 offspring at P7. All tissue and blood samples were collected 2.5 hours following the morning administration of methadone. Data are collapsed across offspring sex. All data are mean ± SEM.

EDDP: 2-ethylidene-1,5-dimethyl-3,3-diphenylpyrrolidine. The limit of quantification for methadone and EDDP detection was 0.1 ng/mL and 0.05 ng/mL in the plasma, respectively, and 0.08 ng/sample and 0.04 ng/sample of placenta and brain for both methadone and EDDP.
