# Supplementary material for: Prenatal methadone exposure disrupts behavioral development and alters motor neuron intrinsic properties and local circuitry
Source: eLife. 2021 Mar 16;10:e66230. doi: 10.7554/eLife.66230 (PMC7993998; doi:10.7554/eLife.66230)
Supplement: Supplementary file 2. — Unpaired t tests, n = 11 (4M:7F) PME, 11 PSE (6M:5F) mice. See Figure 8—figure supplement 1 for visual representation of the data. R, Right; L, Left [file elife-66230-supp2.docx]

**Supplementary File 2 Test Statistics for Volumetric MRI Analysis.**

| Volume of Interest | t ratio | df | p value |
| --- | --- | --- | --- |
| Striatum (R) | 1.58 | 20 | 0.13 |
| Striatum (L) | 0.572 | 20 | 0.57 |
| Cortex | 0.411 | 20 | 0.69 |
| Hippocampus (R) | 0.156 | 20 | 0.88 |
| Hippocampus (L) | 1.11 | 20 | 0.28 |
| Thalamus | 0.224 | 20 | 0.82 |
| Cerebellum | 0.267 | 20 | 0.79 |
| Basal Forebrain-Septum | 1.70 | 20 | 0.11 |
| Hypothalamus | 0.891 | 20 | 0.38 |
| Amygdala (R) | 0.319 | 20 | 0.75 |
| Amygdala (L) | 0.338 | 20 | 0.74 |
| Brain Stem | 2.04 | 20 | 0.055 |
| Superior Colliculus | 0.648 | 20 | 0.52 |
| Olfactory Bulb | 1.57 | 20 | 0.13 |
| Midbrain (R) | 0.746 | 20 | 0.46 |
| Midbrain (L) | 1.19 | 20 | 0.25 |
| Inferior Colliculus (L) | 0.301 | 20 | 0.77 |
| Inferior Colliculus (R) | 0.281 | 20 | 0.78 |

Unpaired t tests, n=11 (4M:7F) PME, 11 PSE (6M:5F) mice. See Supplementary Fig. 8 for visual representation of the data. *R,* Right; *L,* Left
