# Supplementary material for: Prenatal methadone exposure disrupts behavioral development and alters motor neuron intrinsic properties and local circuitry
Source: eLife. 2021 Mar 16;10:e66230. doi: 10.7554/eLife.66230 (PMC7993998; doi:10.7554/eLife.66230)
Supplement: Supplementary file 3. — Resting membrane potential evaluated with no applied current, all other properties evaluated with current applied to hold the membrane potential near minus 70 mV. Data were not collapsed on sex as analyses revealed some main-effects of sex. Data presented as mean ± SEM. F statistics (df = 1,65) are presented in the final column with significant results bolded (*p<0.05). n = 10 PME mice (6M:4F), 30 cells (13M:17F) and n = 9 PSE mice (5M:4F), 23 cells (11M:12F). [file elife-66230-supp3.docx]

**Supplementary File 3 Intrinsic Properties of L5 M1 Neurons.**

|  | **PME-Female** | **PSE-Female** | **PME-Male** | **PSE-Female** | **F Statistics:**  **Interaction**  **Sex**  **Exposure** |
| --- | --- | --- | --- | --- | --- |
| **Resting membrane potential (mV)** | -63.1 ± 1.12 | -64.0 ± 1.02 | -63.2 ± 1.62 | -63.7 ± 1.24 | F=0.0226, p=0.88  F=0.00564, p=0.94  F=0.277, p=0.60 |
| **Holding current (pA)** | -100 ± 16.6 | -81.2 ± 17.1 | -107 ± 27.3 | -86.9 ± 19.7 | F=0.000955, p=0.98  F=0.091, p=0.76  F=0.855, p=0.36 |
| **Input resistance (Mohm)** | 71.6 ± 3.15 | 86.6 ± 5.45 | 75.2 ± 5.74 | 79.9 ± 3.57 | F=1.35, p=0.25  F=0.117, p=0.73  **F =4.93, p=0.030*** |
| **Voltage Threshold (mV)** | -38.4 ± 0.75 | -37.2 ± 0.859 | -38.5 ± 0.900 | -39.1 ± 0.840 | F=1.06, p=0.31  F=1.31, p=0.26  F=0.118, p=0.73 |
| **Current Threshold (pA)** | 223 ± 14.5 | 196 ± 2.0 | 237 ± 21.5 | 230 ± 13.6 | F=0.354, p=0.55  F=2.04, p=0.16  F=1.02, p=0.32 |
| **AP half-width (milliseconds)** | 7.76 e^-4^ ± 1.99 e^-5^ | 8.22 e^-4^ ± 1.90 e^-5^ | 7.68 e^-4^ ± 2.16 e^-5^ | 7.50 e^-4^ ± 1.67 e^-5^ | F=2.28, p=0.14  F=3.56, p=0.063  F=0.436, p=0.51 |
| **Tau (milliseconds)** | 2.46 e^-3^ ± 2.26 e^-4^ | 2.46 e^-3^ ± 1.86 e^-4^ | 2.21 e^-3^ ± 1.66 e^-4^ | 2.21 e^-3^ ± 2.20 e^-4^ | F~0, p>0.99  F=0.111, p=0.74  F~0, p>0.99 |
| **FI Slope (Hz/pA)** | 0.082 ± 0.003 | 0.086 ± 0.004 | 0.081 ± 0.005 | 0.086 ± 0.002 | F=0.00942, p=0.92  F=0.0233, p=0.88  F=1.39, p=0.24 |
| **Voltage Sag (%)** | 49.1 ± 2.55 | 40.8 ± 3.09 | 46.3 ± 4.08 | 39.5 ± 3.37 ­­ | F=0.0507, p=0.82  F=0.379, p=0.54  **F=5.14, p=0.027*** |
| **Voltage Overshoot (%)** | 49.1 ± 2.27 | 41.1 ± 2.47 | 42.1 ± 3.31 | 39.5 ± 3.12 | F=0.881, p=0.35  F=2.24, p=0.14  F=3.40, p=0.070 |
| **Fast after-hyperpolarization (mV)** | -2.70 ± 0.562 | -3.63 ± 0.710 | -4.62 ± 1.24 | -3.20 ± 0.558 | F=2.15, p=0.15  F=0.863, p=0.36  F=0.0933, p=0.76 |
| **Medium after-hyperpolarization (mV)** | -12.0 ± 0.917 | -11.4 ± 0.939 | -7.48 ± 2.44 | -9.15 ± 1.00 | F=0.601, p=0.44  **F=5.50, p=0.022***  F=0.153, p=0.70 |
| **Afterdepolarization (mV)** | 24.4 ± 5.81 | 10.3 ± 6.28 | 12.1 ± 5.66 | 19.4 ± 7.81 | F=2.51, p=0.12  F0.0569, p=0.81  F=0.252, p=0.62 |
| **Height (mV)** | 83.7 ± 1.37 | 85.6 ± 0.983 | 86.9 ± 1.30) | 87.5 ± 1.65 | F=0.192, p=0.66  F=2.96, =0.090  F=0.711, p=0.40 |

Resting membrane potential evaluated with no applied current, all other properties evaluated with current applied to hold the membrane potential near minus 70 mV. Data were not collapsed on sex as analyses revealed some main-effects of sex. Data presented as mean ± SEM. F statistics (df = 1,65) are presented in the final column with significant results bolded (*p<0.05). n=10 PME mice (6M:4F), 30 cells (13M:17F) and n=9 PSE mice (5M:4F), 23 cells (11M:12F).
