# Supplementary material for: Prenatal methadone exposure disrupts behavioral development and alters motor neuron intrinsic properties and local circuitry
Source: eLife. 2021 Mar 16;10:e66230. doi: 10.7554/eLife.66230 (PMC7993998; doi:10.7554/eLife.66230)
Supplement: Transparent reporting form [file elife-66230-transrepform.docx]

***eLife’s* transparent reporting form**

We encourage authors to provide detailed information *within their submission* to facilitate the interpretation and replication of experiments. Authors can upload supporting documentation to indicate the use of appropriate reporting guidelines for health-related research (see [EQUATOR Network](http://www.equator-network.org/%20)), life science research (see the [BioSharing Information Resource](https://biosharing.org/)), or the [ARRIVE guidelines](http://www.plosbiology.org/article/info:doi/10.1371/journal.pbio.1000412) for reporting work involving animal research. Where applicable, authors should refer to any relevant reporting standards documents in this form.

If you have any questions, please consult our Journal Policies and/or contact us: [editorial@elifesciences.org](mailto:editorial@elifesciences.org).

**Sample-size estimation**

- You should state whether an appropriate sample size was computed when the study was being designed
- You should state the statistical method of sample size computation and any required assumptions
- If no explicit power analysis was used, you should describe how you decided what sample (replicate) size (number) to use

Please outline where this information can be found within the submission (e.g., sections or figure legends), or explain why this information doesn’t apply to your submission:

Given the exploratory nature of the study, no power calculations were performed in advance. Instead, sample sizes were carefully determined primarily based on preliminary data from pilot studies and also through rigorous literature searches.

**Replicates**

- You should report how often each experiment was performed
- You should include a definition of biological versus technical replication
- The data obtained should be provided and sufficient information should be provided to indicate the number of independent biological and/or technical replicates
- If you encountered any outliers, you should describe how these were handled
- Criteria for exclusion/inclusion of data should be clearly stated
- High-throughput sequence data should be uploaded before submission, with a private link for reviewers provided (these are available from both GEO and ArrayExpress)

Please outline where this information can be found within the submission (e.g., sections or figure legends), or explain why this information doesn’t apply to your submission:

For data on weights, lengths, and bone measures offspring were utilized from 9 or more litters per exposure group across 2 cohorts. For immunostaining and MRI imaging offspring were utilized from 4 or more litters per exposure group. For electrophysiology related studies, offspring were utilized from 10 or more litters per exposure group across 3 cohorts of animals. For behavioral studies, offspring were utilized from 4 or more litters per exposure group to minimize potential litter effects.

No statistical outliers were encountered or removed; however, the offspring of dams which experienced obstructed labor were not included in any follow up studies. Data exclusions for electrophysiology, mapping, and morphology experiments are extensively described in the methods sections (See "Data Exclusion Criteria" in *Methods*).

**Statistical reporting**

- Statistical analysis methods should be described and justified
- Raw data should be presented in figures whenever informative to do so (typically when N per group is less than 10)
- For each experiment, you should identify the statistical tests used, exact values of N, definitions of center, methods of multiple test correction, and dispersion and precision measures (e.g., mean, median, SD, SEM, confidence intervals; and, for the major substantive results, a measure of effect size (e.g., Pearson's r, Cohen's d)
- Report exact p-values wherever possible alongside the summary statistics and 95% confidence intervals. These should be reported for all key questions and not only when the p-value is less than 0.05.

Please outline where this information can be found within the submission (e.g., sections or figure legends), or explain why this information doesn’t apply to your submission:

The statistical tests performed are described in the *Methods* and in brief before statistics are reported the *Results* section. Furthermore, each statistical comparison is stated in figure legends as well. Sample sizes are provided in figure legends. Data are presented as box and whisker plots with whiskers representing the minimum and maximum values or mean +/- SEM for repeated measures (this is also described in each figure legend. Pearson’s r was reported for correlations in Figure 2, but no other effect sizes were reported. All summary statistics and exact p-values are reported for all analyses

(For large datasets, or papers with a very large number of statistical tests, you may upload a single table file with tests, Ns, etc., with reference to sections in the manuscript.)

**Group allocation**

- Indicate how samples were allocated into experimental groups (in the case of clinical studies, please specify allocation to treatment method); if randomization was used, please also state if restricted randomization was applied
- Indicate if masking was used during group allocation, data collection and/or data analysis

Female C57BL/6J mice (Jackson) were randomly assigned to treatment groups. Offspring were randomly assigned to treatment groups among the various experiments (behavior, immunohistochemistry, electrophysiology, etc.) with careful attention to include offspring from at least 4 or more litters per treatment. Bone data was collected using a SkyScan 1172 (Bruker) and brain MRI data was collected using a 9.4 T Bruker system (Bruker BioSpin). Noldus EthoVision XT software was used to tracking locomotor activity and DeepSqueak (deep learning-based system for detection and analysis of ultrasonic vocalizations; see methods) for detecting USVs in the open field. Startle responses were collected using SR-LAB Startle Response System (SD Instruments). Ephus software (http://scanimage.vidriotechnologies.com/display/ephus/Ephus) was used for hardware control and data collection for all electrophysiology experiments. NIH ImageJ software was used to quantify immunostaining data. For electrophysiology and neuron morphology, Matlab (R2012a, R2015b) and Neurolucida 64-bit , Neurolucida 360, and Neurolucida Explorer (MBF Bioscience) were used. All remaining analyses were completed in GraphPad Prism 8.2.1. All experimenters were blinded to exposure group during data collection and processing for all completed studies.

Please outline where this information can be found within the submission (e.g., sections or figure legends), or explain why this information doesn’t apply to your submission:

**Additional data files (“source data”)**

- We encourage you to upload relevant additional data files, such as numerical data that are represented as a graph in a figure, or as a summary table
- Where provided, these should be in the most useful format, and they can be uploaded as “Source data” files linked to a main figure or table
- Include model definition files including the full list of parameters used
- Include code used for data analysis (e.g., R, MatLab)
- Avoid stating that data files are “available upon request”

Please indicate the figures or tables for which source data files have been provided:

Supplementary Tables and Figures are provided which support the main figures of the text. Source data is provided for all analyses completed
